# Pregnancy vs. Postpartum Breast Cancer: Distinct Tumor Biology and Survival Trends in a Contemporary Cohort

**DOI:** 10.3390/cancers17244031

**Published:** 2025-12-18

**Authors:** Elena Jane Mason, Alba Di Leone, Beatrice Carnassale, Antonio Franco, Cristina Accetta, Sabatino D’Archi, Flavia De Lauretis, Federica Gagliardi, Elisabetta Gambaro, Marzia Lo Russo, Stefano Magno, Francesca Moschella, Federica Murando, Maria Natale, Alejandro Martin Sanchez, Lorenzo Scardina, Marta Silenzi, Alessandra Fabi, Ida Paris, Antonella Palazzo, Armando Orlandi, Fabio Marazzi, Angela Santoro, Paolo Belli, Giacomo Corrado, Patrizia Frittelli, Gianluca Franceschini

**Affiliations:** 1 Breast Surgery Unit, Ospedale Isola Tiberina—Gemelli Isola, 00186 Rome, Italy; 2 Breast Surgery Unit, Department of Woman and Child’s Health and Public Health Sciences, Fondazione Policlinico Universitario A. Gemelli IRCCS, 00168 Rome, Italyelisabetta.gambaro@dr.com (E.G.); stefano.magno@policlinicogemelli.it (S.M.); maria.natale@policlinicogemelli.it (M.N.); martin.sanchez@policlinicogemelli.it (A.M.S.); gianluca.franceschini@policlinicogemelli.it (G.F.); 3 Precision Medicine in Breast Cancer Unit, Department of Woman and Child’s Health and Public Health Sciences, Scientific Directorate, Fondazione Policlinico Universitario A. Gemelli IRCCS, 00168 Rome, Italy; 4 Division of Gynecologic Oncology, Department of Woman and Child’s Health and Public Health Sciences, Fondazione Policlinico Universitario A. Gemelli IRCCS, 00168 Rome, Italy; 5 Unit of Oncology, Comprehensive Cancer Centre, Fondazione Policlinico Universitario A. Gemelli IRCCS, 00168 Rome, Italy; antonella.palazzo@policlinicogemelli.it (A.P.);; 6 UOC Oncological Radiotherapy, Department of Diagnostic Imaging, Radiation Oncology and Haematology, Fondazione Policlinico Universitario A. Gemelli IRCCS, 00168 Rome, Italy; 7 Pathology Unit, Department of Woman and Child’s Health and Public Health Sciences, Fondazione Policlinico Universitario Agostino Gemelli IRCCS, 00168 Rome, Italy; 8 Department of Bioimaging, Radiation Oncology and Hematology, UOC di Radiologia, Fondazione Policlinico Universitario A. Gemelli IRCSS, Largo A. Gemelli 8, 00168 Rome, Italy; 9 Department of Medical and Surgical Sciences, Catholic University of the Sacred Heart, 00168 Rome, Italy

**Keywords:** pregnancy-associated breast cancer, postpartum breast cancer, clinical presentation, tumor biology, survival outcomes

## Abstract

**Background**: Pregnancy-associated breast cancer (PABC), defined as breast cancer diagnosed during pregnancy or within one year postpartum, is a unique and clinically challenging entity. Evidence suggests that tumors diagnosed during pregnancy (PrBC) and postpartum (PPBC) may differ in biology and prognosis. This study compares clinical features, treatment patterns and outcomes between PrBC and PPBC. **Methods**: We performed a retrospective analysis of 76 women diagnosed with PABC from January 2000 to June 2023 across two tertiary centers. Patients were classified according to ESMO guidelines as PrBC (*n* = 41) or PPBC (*n* = 35). Clinical presentation, tumor characteristics, treatment approaches and survival outcomes were evaluated. Overall survival (OS) and disease-free survival (DFS) were estimated using Kaplan–Meier analysis and compared with log-rank tests. **Results**: A total of 76 patients with PABC were included (41 PrBC, 35 PPBC; median age 37 years). Most tumors were high-grade invasive ductal carcinomas, with Luminal B predominant in PrBC and triple-negative breast cancer (TNBC) in PPBC. Locally advanced disease was common (axillary involvement 52%; de novo metastases 9%). Surgery was performed in most cases, with breast conservative surgery (BCS) more frequent in PrBC and mastectomy in PPBC; 46% received neoadjuvant chemotherapy. At median follow-up of 68 months, 7.9% of patients had died and 29% experienced recurrence. Oncologic outcomes were similar between subgroups, with a trend in favor of PrBC. Pregnancy continuation did not adversely affect outcomes. **Conclusions**: PrBC and PPBC display heterogeneous clinical presentations with a trend toward more favorable outcomes in PrBC. These findings support the need for tailored counseling, individualized management and research designs that differentiate between PrBC and PPBC.

## Simple Summary

Pregnancy-associated breast cancer (PABC) is often considered a single entity, but emerging evidence suggests that tumors diagnosed during pregnancy may differ from those identified within the first year postpartum. In this multicenter study of 76 women, we compared clinical presentation, tumor characteristics, treatment patterns and outcomes between pregnancy-diagnosed breast cancer (PrBC) and postpartum breast cancer (PPBC). Our findings indicate that PPBC frequently exhibits aggressive pathological features, and continuing pregnancy does not appear to worsen survival. These results highlight the clinical heterogeneity within PABC and support the need for tailored counseling, individualized management and research designs that differentiate between PrBC and PPBC.

## 1. Introduction

Pregnancy-associated breast cancer (PABC) is defined as breast cancer diagnosed during pregnancy or within one year after childbirth [1]. Its incidence is rising, likely due to increasing rates of breast cancer in premenopausal women and the trend of delayed childbearing [2,3,4].

Traditionally, PABC has been considered to carry a worse prognosis compared with non-pregnancy-related breast cancer [5,6,7,8,9,10]. However, it is increasingly recognized as a heterogeneous entity. In particular, growing evidence suggests biological and clinical differences between breast cancer diagnosed during pregnancy (PrBC) and postpartum breast cancer (PPBC) [11,12,13].

The postpartum period may represent a biologically vulnerable window, potentially influencing tumor progression and prognosis [14,15]. Distinguishing between PrBC and PPBC can be challenging, mainly when symptom onset occurs near delivery, often leading to diagnostic delays. Recent mechanistic studies have shown that the postpartum breast undergoes a distinct involution process that temporarily creates a pro-tumorigenic microenvironment. This period is characterized by extensive extracellular matrix remodeling with increased deposition of fibrillar collagen and heightened COX-2 activity, recruitment of myeloid-derived immune cells and local immunosuppression, and enhanced lymphangiogenesis and stromal remodeling [16,17]. These changes may facilitate tumor invasion and metastatic potential. In contrast, cancers diagnosed during pregnancy arise in a hormonally driven, immune-tolerant environment. Molecular profiling studies further indicate that PPBC exhibits distinct transcriptional features associated with more aggressive behavior [18,19]. These biological differences provide a rationale for evaluating PrBC and PPBC as separate clinical subgroups.

This study aims to compare PrBC and PPBC diagnosed within one year of childbirth, focusing on differences in clinical presentation, tumor characteristics, treatment patterns, and survival outcomes. By clarifying these distinctions, we aim to determine whether these subgroups should be regarded as separate entities in clinical practice and research.

## 2. Materials and Methods

### 2.1. Study Design and Ethical Approval

This retrospective observational study was conducted in accordance with the ethical principles of the Declaration of Helsinki and was approved by the Regional Ethics Committee (Comitato Etico Territoriale Lazio Area 3, Protocol ID: 7324). Data were collected from two tertiary referral centers specializing in both breast cancer and obstetric care: Fondazione Policlinico Universitario Agostino Gemelli IRCCS (FPG) and Ospedale Isola Tiberina—Gemelli Isola (OIT).

We included all women discussed in multidisciplinary team meetings (MDMs) for the management of PABC, defined as breast cancer diagnosed during pregnancy or within one year after delivery [1]. According to ESMO consensus guidelines, patients were stratified into two subgroups: PrBC diagnosed during gestation and PPBC diagnosed within 12 months of childbirth [11]. Patients diagnosed after miscarriage or elective abortion were excluded.

The study population included women treated at FPG from 1 January 2000 to 30 June 2023, and at OIT from 1 January 2016 to 30 June 2023. Given the rarity of PABC, no a priori power calculation was feasible; therefore, all analyses were considered exploratory.

### 2.2. Clinical Management

Due to the retrospective nature of the study, clinical management reflected best practices at the time of treatment. Most patients presented with a palpable breast mass and underwent ultrasound-guided core needle biopsy. Axillary staging was performed using axillary ultrasound. Mammography was selectively used in pregnant patients when necessary for local staging, with appropriate fetal shielding. Breast magnetic resonance imaging (MRI) was not performed in pregnant or lactating women at the time of diagnosis.

All cases were reviewed in MDMs involving oncologists, breast surgeons, radiologists and obstetricians [20]. Neoadjuvant chemotherapy (NACT) was offered to high-risk PrBC patients after the first trimester. Radiotherapy (RT) was deferred until after delivery. Surgical planning aimed to replicate standard care for non-pregnant patients as closely as possible. Breast-conserving surgery was generally discouraged during the first trimester due to institutional guidelines recommending initiation of adjuvant RT within 20 weeks of surgery [21]. Immediate breast reconstruction was routinely offered when mastectomy was indicated.

In PrBC cases, a gynecologist-obstetrician attended all MDMs, collaborating closely with the oncologic team to minimize obstetric complications and avoid preterm birth. Patients received structured counseling regarding pregnancy continuation and were informed that current evidence does not support a survival benefit from pregnancy termination.

### 2.3. Clinical Outcomes

The primary outcome was overall survival (OS) defined as the time from diagnosis to death from any cause. Secondary outcomes included disease-free survival (DFS), distant disease-free survival (DDFS) and differences in tumor characteristics and clinical presentation between PrBC and PPBC groups. A tertiary outcome within the PrBC group assessed survival and recurrence differences between patients who continued pregnancy and those who experienced pregnancy loss.

### 2.4. Statistical Analysis

Statistical analyses were performed using SPSS (version 29.0, MacOS). OS, DFS, and DDFS were estimated using the Kaplan–Meier method and compared with the log-rank test. Continuous variables are reported as mean (median; interquartile range), and categorical variables as frequencies and percentages. Group comparisons were conducted using the Chi-square test or Fisher’s exact test for categorical variables and the Wilcoxon signed-rank test for non-normally distributed continuous data. Normality was assessed using the Shapiro–Wilk test. A *p*-value < 0.05 was considered statistically significant. All tests were two-tailed. An exploratory multivariable Cox proportional hazards model was used to adjust DFS for age at diagnosis, stage, tumor subtype and nodal status. Given the limited number of events, the analysis was considered exploratory. A Cox model for OS was not performed because the small number of events precluded a meaningful adjusted analysis.

## 3. Results

### 3.1. Patients Characteristics

A total of 76 patients met the inclusion criteria: 41 (53.9%) were diagnosed during PrBC and 35 (46.1%) within the PPBC. In the PrBC group, diagnoses were distributed across trimesters as follows: first trimester in 16 cases (21.1%), second trimester in 11 cases (14.5%), and third trimester in 14 cases (18.4%). The median age at diagnosis was 37 years (IQR 34–41) in both groups.

Most patients presented with locally advanced disease. Axillary lymph node involvement was observed in 51.6% of cases, and 7 patients (9.2%) had de novo metastatic disease at diagnosis. The majority of tumors were high-grade ductal invasive carcinomas (DCI), with Luminal B being the most frequent molecular subtype (40.8%), followed by triple-negative breast cancer (TNBC). Symptom onset dates were not uniformly available in this retrospective cohort; therefore, diagnostic delay could not be formally assessed.

Patient and disease characteristics are detailed in Table 1.

### 3.2. Treatments Characteristics

Treatment details are summarized in Table 2. Surgical management of the breast and axilla was performed in the majority of patients. Surgery occurred during pregnancy in 26% of PrBC patients, with no notable variation by trimester. Breast-conserving surgery (BCS) was the most common procedure (54.8%), while 33 patients (45.2%) underwent mastectomy, usually followed by immediate breast reconstruction (IBR, 87.8%).

Axillary surgery was performed in 70 patients; axillary lymph node dissection was required in 44.3% of patients due to nodal involvement.

NACT was administered to 46.1% of patients, reflecting the high proportion of advanced-stage disease. Among the 35 patients receiving NACT, 10 initiated treatment during pregnancy. The pathological complete response (pCR) rate was 37.1%, with an additional 22.9% achieving a major pathological response (residual invasive disease < 5 mm).

### 3.3. PrBC vs. PPBC: Clinical and Pathological Differences

Comparison of PrBC and PPBC subgroups revealed notable trends:

- Tumor subtypes: Luminal B tumors predominated in PrBC, whereas TNBC was more frequent in PPBC. HER2-positive tumors accounted for ~25% in both groups. Luminal A tumors and ductal carcinoma in situ (DCIS) were rare.
- Histology: High-grade tumors were common in both cohorts, especially in PPBC (>70%).
- Stage distribution: Stage I disease was rare overall but twice as common in PrBC. Stage II tumors accounted for >65% of PPBC cases.

Treatment patterns differed slightly between groups:

- Mastectomy was more frequent in PPBC, while BCS was more commonly performed in PrBC.
- PPBC patients were more likely to receive NACT, likely due to higher TNBC prevalence and fewer pregnancy-related treatment limitations.
- NACT response rates were higher in PPBC, possibly reflecting differences in tumor subtype distribution or treatment delivery constraints due to ongoing pregnancy.

Although most differences were not statistically significant, they suggest potential distinctions between PrBC and PPBC that warrant investigation in larger cohorts (Table 3).

### 3.4. Survival Analysis

At a median follow-up of 68 months for OS (IQR 30–89) and 57 months for DFS (IQR 28–80), 6 of the 76 patients (7.9%) had died, mostly within the first three years following diagnosis (range 14–101 months). Among the 72 patients who achieved a disease-free state, recurrence occurred in 21 cases (29.2%): 12 local (15.8%) and 14 systemic (19.2%) events were recorded.

**Figure 1:**
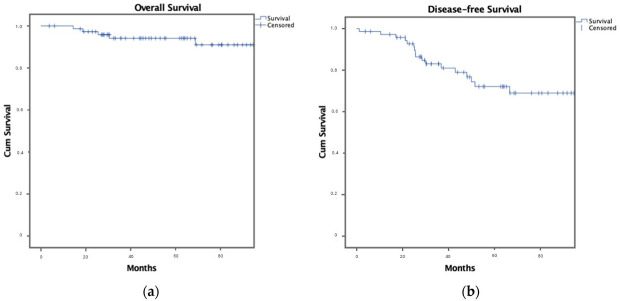
Kaplan–Meier curves illustrating (**a**) Overall Survival and (**b**) Disease-Free Survival in patients with Pregnancy-Associated Breast Cancer (PABC). Number of patients included: *n* = 76. All stage IV patients were included in the OS analysis. DFS analysis excluded 4 stage IV patients who did not achieve disease-free status.

**Figure 2:**
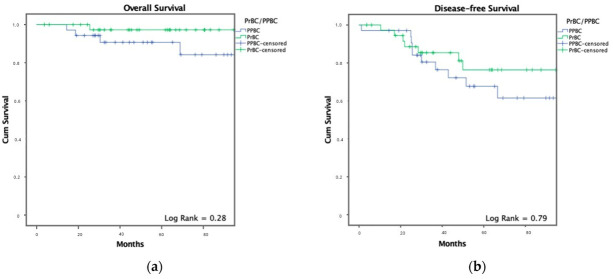
Kaplan–Meier curves illustrating (**a**) Overall Survival and (**b**) Disease-Free Survival in patients with breast cancer diagnosed during pregnancy (PrBC—green) versus the postpartum period (PPBC—blue). Number of patients included: *n* = 76. All stage IV patients were included in the OS analysis. DFS analysis excluded 4 stage IV patients who did not achieve disease-free status.

While not statistically significant, survival analysis demonstrated a trend favoring the PrBC group. Death occurred in 2 of 41 PrBC patients (4.9%) versus 4 of 35 PPBC patients (11.4%). Recurrence affected 10 of 38 PrBC patients (26.3%) and 11 of 34 PPBC patients (32.4%). Kaplan–Meier curves illustrating OS and DFS comparisons are presented in Figure 1 and Figure 2, and data on recurrence patterns are summarized in Table 4.

An exploratory Cox proportional hazards model was performed to evaluate whether the apparent survival differences between PrBC and PPBC persisted after adjusting for age at diagnosis, stage, subtype and nodal status. In this model, PPBC was not independently associated with worse DFS (HR 1.06, *p* = 0.91). None of the covariates reached statistical significance, although nodal positivity showed the strongest association with recurrence (HR 2.20, *p* = 0.16). Given the limited number of events (*n* = 19), these results should be interpreted with caution and considered hypothesis-generating.

### 3.5. Pregnancy Continuation vs. Termination

In the PrBC group, most patients continued pregnancy to term. Nine patients experienced pregnancy termination, either due to miscarriage or elective abortion. Treatment strategies were carefully adapted across all trimesters to ensure maternal and fetal safety.

**Figure 3:**
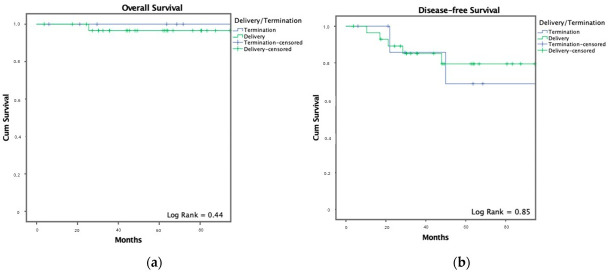
Kaplan–Meier curves depicting (**a**) Overall Survival and (**b**) Disease-Free Survival among patients diagnosed with breast cancer during pregnancy (PrBC), comparing individuals who continued the pregnancy to term with those who experienced pregnancy interruption. Number of patients included: *n* = 41. All stage IV patients were included in the OS analysis. DFS analysis excluded 2 stage IV patients who did not achieve disease-free status.

No survival benefit was observed for pregnancy termination: OS and DFS were comparable between patients who continued pregnancy and those who did not (Figure 3).

## 4. Discussion

PABC remains a complex clinical entity due to the interplay between the physiological changes during pregnancy and the challenges of oncologic management. In our retrospective analysis of 76 patients diagnosed with PABC between 2000 and 2023, we observed a local recurrence rate of 9.2% and a systemic recurrence rate of 18.4%. These figures are consistent with previously reported recurrence rates in high-risk early breast cancer populations and likely reflect the aggressive tumor biology often associated with PABC [22,23,24].

In this cohort, we examined the clinical and biological characteristics of breast cancers diagnosed during pregnancy (PrBC, *n* = 41) and within the first year postpartum (PPBC, *n* = 35). Patients with PrBC were more likely to present with Luminal B tumors (48.8% vs. 31.4% in PPBC) and earlier-stage disease, whereas PPBC showed a higher prevalence of TNBC (31.4% vs. 17.1% in PrBC) and high-grade tumors [25,26,27,28]. The higher prevalence of TNBC in PPBC observed in our cohort is consistent with mechanistic models of postpartum involution, which describe increased COX-2 activity, collagen remodeling, and immune-cell recruitment that may transiently favor more aggressive tumor phenotypes [18,29,30], although the small sample size and observational design preclude any causal inference.

The majority of patients presented with locally advanced disease, with 51.6% exhibiting nodal involvement and 9.2% diagnosed with de novo metastatic disease. At a median follow-up of 68 months, 6 patients (7.9%) had died, and 21 (29.2%) experienced recurrence. Although differences in OS (4.8% mortality in PrBC vs. 11.4% in PPBC; *p* = 0.28) and DFS (26.3% recurrence in PrBC vs. 32.4% in PPBC; *p* = 0.79) did not reach statistical significance, there was a trend toward better outcomes in the PrBC group. Systemic recurrence was more frequent in PPBC than in PrBC, a finding in line with previous studies suggesting that the postpartum microenvironment may promote more invasive or metastatic tumor phenotypes [18,29,30]. However, these differences were not statistically significant and event numbers were small, therefore data should be interpreted cautiously. To account for potential confounding, we conducted an exploratory multivariable Cox analysis adjusting for age, stage, subtype and nodal status. As expected, given the small number of DFS events, no variable reached statistical significance and the confidence intervals were wide. Importantly, PrBC versus PPBC status was not independently associated with recurrence risk after adjustment. These findings support the descriptive nature of our results and reinforce the need for larger multicenter studies with adequate statistical power.

Treatment patterns reflected both tumor biology and logistical considerations. The higher use of NACT in PPBC likely reflects the higher frequency of more aggressive tumor subtypes in this cohort, and the absence of pregnancy-related treatment constraints. Interestingly, pCR rates were numerically higher in PPBC, particularly among TNBC patients. Administration of chemotherapy postpartum was less constrained in this cohort, allowing for optimal dosing and scheduling, which may partially explain these trends. Prior studies have also suggested increased chemosensitivity in PPBC, particularly in highly proliferative subtypes [31,32], although our data were not powered to assess this directly. Importantly, pregnancy termination did not confer a survival benefit, consistent with current guidelines discouraging elective abortion solely for oncologic reasons [1,33,34]. This supports the feasibility and safety of treating breast cancer during pregnancy, particularly after the first trimester, within a multidisciplinary framework.

The strengths of this study include a relatively large sample size for this rare condition and a multicentric design involving high-volume breast centers, which enhances data consistency and generalizability. Limitations include its retrospective nature, modest subgroup sample sizes and possible underrepresentation of patients presenting with metastatic disease, who may have been managed primarily in medical oncology rather than surgical multidisciplinary meetings. This may have influenced stage distribution at diagnosis and could lead to an underestimation of recurrence events. The 23-year inclusion period may represent an additional limitation, as diagnostic modalities, pathological reporting standards, and systemic treatments—particularly access to HER2-targeted agents and routine genetic testing—have evolved substantially over time. However, this reflects the real-world nature of PABC care over two decades, and importantly, the distribution of PrBC and PPBC cases did not cluster in specific time intervals, reducing the likelihood of systematic bias between the two subgroups.

Finally, although emerging evidence suggests that postpartum involution may influence breast cancer biology for several years after childbirth [9], our study applied the conventional ≤12-month definition of PPBC used in clinical guidelines and most of the prior literature. We acknowledge that this may lead to misclassification in the PrBC group of some cases of biologically PPBCs postpartum tumors diagnosed beyond 12 months, which reflects the limitation of the current definition rather than of the dataset itself. Future studies should stratify patients by parity and timing of prior pregnancies to better define the impact of recent gestation on tumor characteristics and prognosis, enabling more precise classification and personalized management strategies.

## 5. Conclusions

This study describes clinical and biological differences between breast cancers diagnosed during pregnancy and those diagnosed in the first year postpartum. PrBC in our cohort was more often associated with Luminal B tumors and earlier-stage disease, whereas PPBC showed a higher prevalence of triple-negative and high-grade tumors. Survival and recurrence outcomes did not differ significantly between groups, although we noted a trend toward improved OS and DFS in the PrBC group. Crucially, continuation of pregnancy did not negatively impact survival outcomes, supporting the safety of administering standard oncologic treatments during gestation.

Prospective, multicenter studies are warranted to confirm these observations and guide future clinical practice, potentially informing tailored treatment strategies according to the timing of breast cancer diagnosis relative to pregnancy.

## Figures and Tables

**Table 1:** Patient and tumor characteristics for pregnancy-associated breast cancer (PABC).

Characteristic	Category	*n* (%)
Age at diagnosis (years)	Median (range)	37 (25–47)
Previous pregnancy	Yes	43 (66.2%)
	No	22 (33.8%)
Genetic testing ^†^	BRCA1	7 (12.7%)
	BRCA2	4 (7.3%)
	PALB2	1 (1.8%)
	None/Not tested	43 (78.2%)
Pregnancy outcome	Childbirth	66 (86.8%)
	Termination (any cause)	10 (13.2%)
Clinical tumor stage	Tis	2 (3.0%)
	T1b	6 (9.0%)
	T1c	19 (28.4%)
	T2	31 (46.3%)
	T3	8 (11.9%)
	T4	1 (1.5%)
Final tumor stage ^†^	pTis	2 (2.9%)
	pT0	13 (18.6%)
	pT1mi	6 (8.6%)
	pT1a	4 (5.7%)
	pT1b	4 (5.7%)
	pT1c	23 (32.9%)
	pT2	13 (18.6%)
	pT3	5 (7.1%)
Clinical nodal status ^†^	N0	29 (48.3%)
	N1	26 (43.3%)
	N2	5 (8.3%)
Final nodal status ^◊^	pN0	38 (56.7%)
	pN1	18 (26.9%)
	pN2	10 (14.9%)
	pN3	1 (1.5%)
Metastatic disease at diagnosis	Yes	7 (9.2%)
	No	69 (90.8%)
Histologic type	Invasive ductal carcinoma	67 (88.2%)
	Invasive lobular carcinoma	3 (3.9%)
	DCIS	2 (2.6%)
	Other	4 (5.3%)
Final disease stage ^†^	0	2 (2.7%)
	I	12 (16.2%)
	IIa	21 (28.4%)
	IIb	18 (24.3%)
	IIIa	12 (16.2%)
	IIIb	1 (1.4%)
	IIIc	1 (1.4%)
	IV	7 (12.2%)
Tumor grade ^◊^	G1	1 (1.4%)
	G2	25 (36.2%)
	G3	43 (62.3%)
Subtype	Luminal A	6 (7.9%)
	Luminal B	31 (40.8%)
	HER2+/ER−	11 (14.5%)
	HER2+/ER+	8 (10.5%)
	Triple negative (TNBC)	18 (23.7%)
	DCIS	2 (2.6%)
Multifocality ^†^	Yes	13 (18.1%)
	No	59 (81.9%)

^†^ Percentages are calculated on available data. Missing data (0–15% across variables) mainly reflected variations in diagnostic practice over the long study period and appeared random, without differences between PrBC and PPBC. ^◊^ Based on final histology.

**Table 2:** Patient and tumor characteristics for pregnancy-associated breast cancer (PABC).

Characteristic ^‡^	Category	*n* (%)
Timing of surgery	First trimester	6 (7.9%)
	Second trimester	6 (7.9%)
	Third trimester	7 (9.2%)
	Postpartum	46 (60.5%)
	Post-termination	8 (10.5%)
	No surgery	3 (3.9%)
Type of breast surgery ^†^	Mastectomy	33 (45.2%)
	– with immediate reconstruction	29 (87.9%)
	– without reconstruction	4 (12.1%)
	Breast-conserving surgery (BCS)	40 (54.8%)
Axillary surgery	Sentinel lymph node biopsy (SLNB)	32 (45.7%)
	Nodal sampling	7 (10.0%)
	Axillary dissection (SLNB+)	8 (11.4%)
	Upfront axillary dissection	23 (32.9%)
Surgery during pregnancy ^†^	Yes	19 (26.0%)
	No	54 (74.0%)
Neoadjuvant chemotherapy (NACT)	Any NACT	35 (46.1%)
	NACT during pregnancy	10 (13.2%)
NACT response ^§^	Complete response	13 (37.1%)
	Major response	8 (22.9%)
	Partial response	11 (31.4%)
	No response	3 (8.6%)

^‡^ Percentages are calculated on available data. Missing data (0–8% across variables) mainly reflected variations in diagnostic practice over the long study period and appeared random, without differences between PrBC and PPBC. ^†^ Among 73 patients who received surgery. ^§^ Among 35 patients who received NACT.

**Table 3:** Differences in presentation, clinical features, and treatment between Pregnancy Breast Cancer (PrBC) and Post-pregnancy breast cancer (PPBC) groups.

Variable	PPBC (*n* = 35)	PrBC (*n* = 41)	*p*-Value
**Subtype**			
TNBC	12 (34.3%)	6 (14.6%)	0.43
HER2+/ER−	5 (14.3%)	6 (14.6%)	
HER2+/ER+	4 (11.4%)	4 (9.8%)	
Luminal B	11 (31.4%)	20 (48.8%)	
Luminal A	2 (5.7%)	4 (9.8%)	
DCIS	1 (2.9%)	1 (2.4%)	
**Tumor grade**			
G1	1 (3.1%)	0	
G2	8 (25.0%)	17 (45.9%)	0.13
G3	23 (71.9%)	20 (54.1%)	
**Stage (final)**			
0/I	5 (14.3%)	9 (23.1%)	0.30
II	23 (65.7%)	16 (41.0%)	
III	5 (14.3%)	9 (23.1%)	
IV	2 (5.7%)	5 (12.8%)	
**Genetic mutation**	6 (23.0%)	6 (20.7%)	0.76
**Prior pregnancy**	24 (77.4%)	19 (55.9%)	0.067
**Type of breast surgery**			
Mastectomy	19 (55.9%)	14 (35.9%)	0.09
BCS	15 (44.1%)	25 (64.1%)	
**Axillary surgery**			
SLNB	16 (47.1%)	16 (44.4%)	0.92
Upfront dissection	10 (29.4%)	13 (36.1%)	
Dissection (SLNB+)	4 (11.8%)	4 (11.1%)	
**Neoadjuvant Chemotherapy (NACT)**	21 (60.0%)	14 (34.1%)	**0.024**
**Response to NACT ^†^**			
Complete response	9 (42.8%)	4 (28.6%)	0.176
Major response	5 (23.8%)	3 (21.4%)	
Partial response	6 (28.6%)	5 (35.7%)	
Non-responder	1 (4.8%)	2 (14.3%)	

^†^ Among patients who received NACT.

**Table 4:** Recurrence patterns in the pregnancy-associated breast cancer (PABC) cohort and in subgroups pregnancy breast cancer (PrBC) and post-pregnancy breast cancer (PPBC).

Recurrence Type	PABC (*n* = 76)	PPBC (*n* = 35)	PrBC (*n* = 41)	*p*-Value ^§^
No recurrence	55 (72.4%)	24 (68.6%)	31 (75.6%)	—
Local recurrence	7 (9.2%)	3 (8.6%)	4 (9.8%)	—
Any systemic recurrence ^†^	14 (18.4%)	8 (22.9%)	6 (14.6%)	0.659

^§^ Comparison between local and systemic recurrence in PrBC and PPBC subgroups. Given small sample sizes, data were analyzed using Fisher’s exact test. ^†^ Includes distant metastases with or without concurrent local recurrence.

## Data Availability

The data presented in this study are available on request from the corresponding author.

## Abbreviations

The following abbreviations are used in this manuscript:

PABC	Pregnancy-associated breast cancer
PrBC	Breast cancer occurring during pregnancy
PPBC	Postpartum breast cancer
OS	Overall survival
DFS	Disease-free survival
DDFS	Distant disease-free survival
MDMs	Multidisciplinary team meetings
TNBC	Triple negative breast cancer
DCI	Ductal invasive carcinoma
DCIS	Ductal in situ carcinoma
NACT	Neoadjuvant chemotherapy
RT	Radiotherapy
BCS	Breast conserving surgery
IBR	Immediate breast reconstruction
pCR	Pathologic complete response
MRI	Magnetic Resonance Imaging
FPG	Fondazione Policlinico Universitario A Gemelli IRCCS
OIT	Ospedale Isola Tiberina—Gemelli Isola
